# Barriers to adherence of posttreatment follow-up after positive primary cervical cancer screening in Ethiopia: a mixed-methods study

**DOI:** 10.1093/oncolo/oyae305

**Published:** 2024-11-18

**Authors:** Rahel Alemayehu, Clara Yolanda Stroetmann, Abigiya Wondimagegnehu, Friedemann Rabe, Adamu Addissie, Eva Johanna Kantelhardt, Muluken Gizaw

**Affiliations:** Department of Epidemiology and Biostatistics, School of Public Health, College of Health Sciences, Addis Ababa University, Addis Ababa, Ethiopia; Global Health Working Group, Institute of Medical Epidemiology, Biometrics and Informatics, Martin-Luther-University Halle-Wittenberg, Halle (Saale), Germany; Global Health Working Group, Institute of Medical Epidemiology, Biometrics and Informatics, Martin-Luther-University Halle-Wittenberg, Halle (Saale), Germany; Department of Epidemiology and Biostatistics, School of Public Health, College of Health Sciences, Addis Ababa University, Addis Ababa, Ethiopia; Global Health Working Group, Institute of Medical Epidemiology, Biometrics and Informatics, Martin-Luther-University Halle-Wittenberg, Halle (Saale), Germany; NCD Working Group, School of Public Health, Addis Ababa University, Addis Ababa, Ethiopia; Global Health Working Group, Institute of Medical Epidemiology, Biometrics and Informatics, Martin-Luther-University Halle-Wittenberg, Halle (SaaleGermany; Department of Epidemiology and Biostatistics, School of Public Health, College of Health Sciences, Addis Ababa University, Addis Ababa, Ethiopia; Global Health Working Group, Institute of Medical Epidemiology, Biometrics and Informatics, Martin-Luther-University Halle-Wittenberg, Halle (Saale), Germany; NCD Working Group, School of Public Health, Addis Ababa University, Addis Ababa, Ethiopia; Global Health Working Group, Institute of Medical Epidemiology, Biometrics and Informatics, Martin-Luther-University Halle-Wittenberg, Halle (Saale), Germany; Department of Gynaecology, Martin-Luther-University Halle-Wittenberg, Halle (Saale), Germany; Department of Epidemiology and Biostatistics, School of Public Health, College of Health Sciences, Addis Ababa University, Addis Ababa, Ethiopia; Global Health Working Group, Institute of Medical Epidemiology, Biometrics and Informatics, Martin-Luther-University Halle-Wittenberg, Halle (Saale), Germany; NCD Working Group, School of Public Health, Addis Ababa University, Addis Ababa, Ethiopia

**Keywords:** cervical cancer screening, adherence to follow-up, barriers, cryotherapy, Ethiopia

## Abstract

**Background:**

Even though it is preventable, cervical cancer contributes significantly to cancer-related mortality among Ethiopian women. Follow-up visits after treatment of precancerous lesions are essential to monitor lesion recurrence. In our previous study, we found a level of adherence to follow-up of 44.7%, but the reasons for low adherence have not been comprehensively explored within the Ethiopian context. This study aimed to identify these reasons by interviewing 167 women who had missed their follow-up appointments as well as 30 health professionals with experience in the field.

**Methods:**

The study employed a mixed-methods approach: Quantitative data were collected through a telephone questionnaire conducted with 167 women who had a positive visual inspection with acetic acid (VIA) and had missed their follow-up appointments. Subsequently, in-depth interviews were conducted with 30 healthcare professionals, and an inductive content analysis was carried out.

**Results:**

In the patient interviews, the reasons given most often were “lack of information about the follow-up” (35; 21.1%), “forgetting the appointment” (30; 18.1%), and “not seeing the need for follow-up” (24; 14.5%). Healthcare professionals identified various reasons such as lack of knowledge, living in a remote area/changing living area, forgetfulness, fear, poor counseling, a shortage of trained healthcare providers to give counseling and follow-up, and reminder-related barriers.

**Conclusion:**

Lack of knowledge, forgetfulness, poor health-seeking behavior, and a lack of reminders were identified as barriers contributing to the low uptake of rescreening. Further interventions should target these by creating community awareness, improving patient counseling, tracing patients in need of follow-up, and making reminder calls or using SMS.

**Implications for Practice**

This study may inform the development of healthcare policies and interventions tailored to address specific challenges faced by patients with visual inspection with acetic acid (VIA)–positive lesions. It has highlighted areas where posttreatment follow-up services can be improved and can help integrate follow-up services more seamlessly into existing healthcare systems. Our results may help in adjustments to be made to interventions based on emerging challenges. By addressing the barriers to posttreatment follow-up after positive VIA screening, healthcare systems can improve the effectiveness of cervical cancer screening programs and reduce the burden of the disease.

## Introduction

In high-resource countries, cervical cancer screening has been shown to reduce the incidence and mortality of the disease.^1-3^ However, in Ethiopia, cervical cancer is still the second leading cause of cancer death among women, with an estimated 7445 new cases and 5338 deaths in 2020.^4^ The Ethiopian Ministry of Health (MoH) recommends cervical cancer screening (CCS) via visual inspection with acetic acid (VIA) every 5 years and intensified screening for women living with HIV (every 2 years). Those with a previous positive screening should rescreen 1 year after treatment.^5^ If precancerous lesions are found, those are usually treated with cryotherapy or thermal ablation, preferably in a single-visit approach.

Approximately 15% of women encounter recurrent or residual precancerous lesions after treatment.^6^ The influence of the HIV status on recurrence rates has been discussed, but while some studies have shown higher recurrence rates in women living with HIV, clear evidence is still lacking.^7-10^

Studies in different settings have indicated high rates of loss to follow-up after screening and treatment of precancerous lesions, ranging from 32% to 80.3%.^11-15^ The level of adherence to follow-up found in our previous study among a cohort of 741 Ethiopian women in Oromia and Addis Ababa was 44.7%.^16^ Due to resource limitations and poor organization of healthcare systems, limited adherence to follow-up continues to be a problem in low- and middle-income countries and is associated with an elevated risk of developing cervical cancer.^17^

Previous studies in other countries have identified various barriers to compliance with follow-up requirements. These include socio-demographic factors such as the women’s educational level, the influence of male companions or partners, financial constraints per transportation to healthcare facilities, and fear of adverse effects such as infertility. Furthermore, facility-related barriers such as staff attitude, cost of service, and inadequate counseling have also emerged.^18-21^

Identifying barriers to adhering to posttreatment follow-up can help policy-makers and program managers address those obstacles through designing effective interventions. This, in turn, may enhance women’s adherence to posttreatment follow-up to prevent the recurrence of precancerous cervical lesions and their progression into invasive cancer. As of now, little is known about barriers associated with nonadherence in Ethiopia. Therefore, this study aimed to fill the gap by exploring barriers to posttreatment follow-up in Addis Ababa and the Oromia region of Ethiopia.

## Methods

### Study design and setting

This study was a continuation of a 2022 study involving 10 health centers located in 4 subcities of Addis Ababa and 4 hospitals in the Oromia region. The results from logbook reviews and phone interviews were detailed in our previous paper, which also provides a comprehensive overview of the methodologies employed for the phone interviews, including questionnaire and sampling procedures.^16^ This paper specifically explores reasons for nonadherence to follow-up from patients. The in-depth interviews (IDIs) focused on health professionals’ experiences with facility and community-related barriers.

### Quantitative data collection and analysis

**Figure 1.**
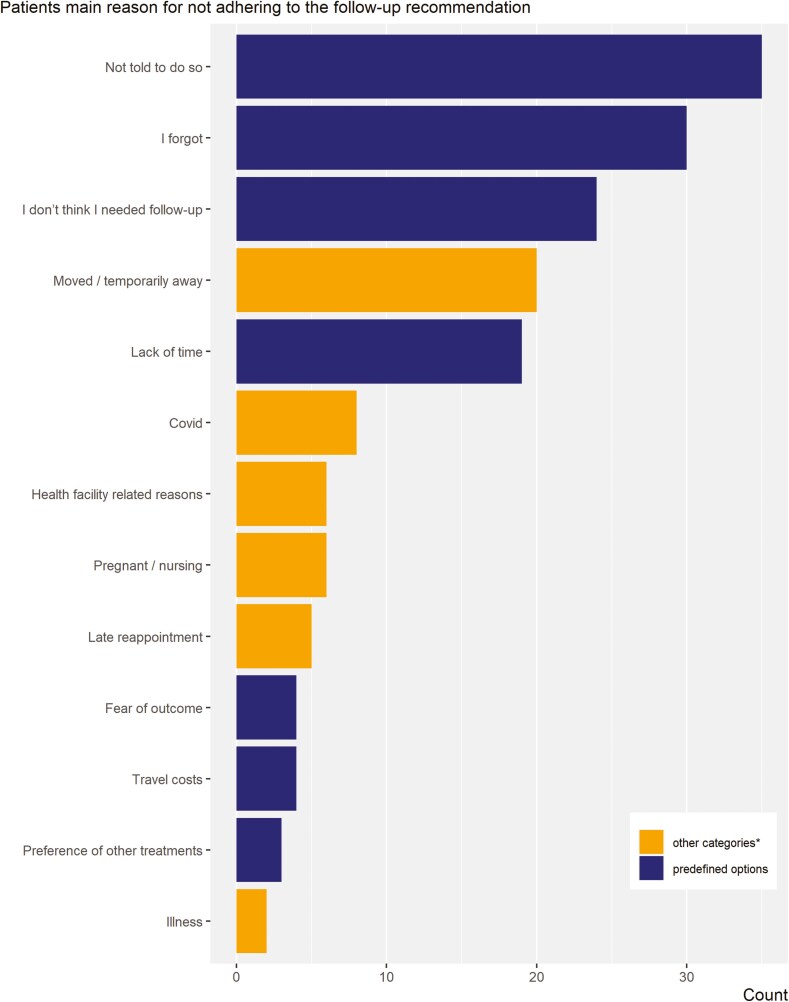
Reasons for not adhering to follow-up appointments as stated by patients (*n* = 166; one answer per patient).

All women with registered phone numbers (574 patients) were invited to participate in a questionnaire-based phone interview, and 399 responded. Of these, 365 received initial treatment of their precancerous lesion. The adherence to post-treatment follow-up among our phone interview participants was 54.2%; the remaining 167 women were questioned about the reasons for nonadherence to follow-up; 166 responded. As part of the quantitative questionnaire-based interview, 9 choices based on our literature review were provided: lack of time, travel costs, “I forgot,” “I didn’t think I needed follow-up,” fear of outcome, unsupportive spouse, “not told to do so,” preference of other treatments, and the option “other” where women could elaborate. The responses to the “other” question were categorized by the 2 principal investigators which led to the result in Figure 1.

### Qualitative data collection and analysis

The qualitative IDIs with 30 healthcare professionals working in the field of CCS were conducted by a team of 2 trained research assistants and 1 of the principal investigators. Our interview partners included medical directors of hospitals, CCS service providers or focal personnel, noncommunicable disease (NCD) team leaders, and maternal and child health coordinators. The interviews were conducted in the local language Amharic, audio-recorded, and supported by field notes. The interview guide (Supplement 1) developed by the principal investigators aimed to explore barriers linked to posttreatment follow-up adherence following CCS.

All the recorded interviews were transcribed and translated into English verbatim. These translated data were uploaded onto qcamap.org^22^ and subjected to coding using inductive content analysis techniques, first led by the principal investigator and then cross-checked by the research team. The content analysis was geared toward synthesizing findings and identifying key themes, aligning with inductive category formation methodology.^23^ The research question was: “What aspects lead to loss of follow-up after treatment of precancerous cervical lesions from a health worker’s perspective?”A coding-recoding evaluation agreement was achieved.

### Ethical approval

Our study adhered to ethical standards with clearance granted by the School of Public Health, Addis Ababa University (Ref.No.SPH/1321/14). All the interviewed participants were informed about the study’s objectives, and their consent to participate was obtained.

## Results

### Patients’ characteristics

In Table 1, an overview of the socio-demographic characteristics of the 166 patients is presented. The median age was 34 years. Almost three-fourths (120, 72.3%) of the interviewed women were currently married, and most (148, 89.2%) had at least 1 child. The majority were either illiterate (32, 19.3%) or had only primary education (48, 28.9%). More than one-third were “housewife/unemployed” (57, 34.3%), while the private sector (42, 25.3%) and government employees (39, 23.5%) also participated. The median self-reported income was 3000 ETB (52.8 USD) per month.

**Table 1.** Socio-demographic characteristics of patients who did not adhere to follow-up (*n* = 166).

Variable	Units	Frequency (%) *n* = 166
Age	≤29 years	35 (21.1%)
	30–34 years	53 (31.9%)
	35–39 years	47 (28.3%)
	≥40 years	31 (18.7%)
	Median age (IQR)	34 years (8)
Marital status	Married	120 (72.3%)
	Divorced	25 (15.1%)
	Single	13 (7.8%)
	Widowed	8 (4.8%)
Parity	0	18 (10.8%)
	1	52 (31.3%)
	2	45 (27.1%)
	3	22 (13.3%)
	>3	29 (17.5%)
Educational Status	Illiterate	32 (19.3%)
	Can read and write	13 (7.8%)
	Primary education	48 (28.9%)
	Secondary education	38 (22.9%)
	College	35 (21.1%)
Occupation	Housewife/unemployed	57 (34.3%)
	Private employee	42 (25.3%)
	Government employee	39 (23.5%)
	Daily laborer	14 (8.4)
	Merchant	9 (5.4%)
	Farmer	1 (0.6%)
	Unknown	4 (2.4%)
Monthly income	Median	3000 ETB (52.8 USD)^a^

^a^Converted using http://www.forbes.com.

### Patients’ reasons for not attending follow-up

The most common predefined reasons selected were not being informed about the follow-up (35, 21.1%) and forgetting the appointment (30, 18.1%). Other common reasons chosen by respondents included not perceiving the follow-up as necessary/feeling healthy (24, 14.5%) and lack of time (19, 11.4%). Among the reasons, participants cited independently included having moved or being temporarily away (20, 12%), which were significant factors in missing appointments (Figure 1).

### Health professionals’ perspectives

Characteristics of the 30 interviewed health professionals are displayed in Table 2. Eighteen (60%) were females, with a median age of 32.5 years. More than three-fourths (23, 76.7%) received special training on CCS.

**Table 2.** Characteristics of interviewed health professionals (*n* = 30).

Variables	Categories	Frequency
Sex	Male	12
	Female	18
Age	≤29 years	5
	30–34 years	14
	35–39 years	5
	≥40 years	6
	Median age (IQR)	32.5 (6)
Work position	CCS focal or service provider	16
	Medical director	3
	NCD team leader	9
	MCH coordinator	2
Work experience on the position in years	≤2 years	17
	3–5 years	9
	6–10 years	4
CCS training	Yes	23
	No	7
Cryotherapy training	Yes	22
	No	8

Abbreviation: MCH: maternal and child health.

Barriers to adhering to follow-up were categorized into 2 distinct groups: patient-related and facility-related barriers (Supplement 2). Patient-related barriers included socioeconomic hindrances, lack of awareness, poor health-seeking behavior, residency-related barriers (like living in a very remote area/changing living area, or difficulty taking public transport to the health facility), forgetfulness, and fear. Facility-related barriers included a shortage of trained healthcare providers, deficient counseling practices, and reminder-related barriers, such as a lack of staff to facilitate reminder calls, no telephone in the CCS unit, frequent change of logbook, and improper documentation of patient data.

### Patient-related barriers

During the IDIs, the participants emphasized socioeconomic factors that impeded women from attending follow-up appointments. They described heavy workloads at home that prevented most women from leaving, with insufficient time for their own health. An NCD team leader portrayed this situation, stating: “Women’s burden in the community is high from performing different activities including child raising, social activity, community engagement, and income-generating activities. So, they are too busy to take the screening and treatment programs” (age 32). Participants discussed the influence of husbands in women’s health decisions, with 1 CCS service provider noting: “[The] husbands’ influence is another factor since most women economically depend on their husbands. So, they have to respect their husbands’ decisions to undergo treatment and follow-up” (age 30). Also, transportation costs were identified as a possible challenge to accessing follow-up services. An NCD team leader pointed out: “They may also have an economic problem such as lack of money for transportation” (age 52).

The geographical distance to the health facility was also considered an impeding factor for both patients and healthcare providers, as illustrated in the following quote: “We try to contact them directly in person by searching for their permanent residence location based on the contact information from the patient’s card collaboratively with the health extension workers. This process works for only those clients whose location is in our woreda[administrative region]” (CCS service provider, age 34). A medical director expressed the belief that women who frequently change their place of residence are at higher risk of missing their follow-up screening, saying: “They may move after screening and treatment. But the permanent residents return for follow-up. One factor for not coming back for their follow-up is being a non-permanent resident” (age 35).

Another prevalent subtheme was the lack of awareness about cervical cancer preventive measures, as expressed by 1 participant: “The biggest barrier on the patient side is the awareness gap, because most of the clients’ awareness about the importance of follow-up is very low” (CCS focal person, age 34). The lack of awareness was particularly problematic because women undergoing screening typically remain asymptomatic and perceive themselves as healthy. The absence of noticeable symptoms and the limited understanding of the screening’s preventive effect were recognized as significant barriers by various interviewees, expressed in the following statements: “Especially those who come from rural areas, if they don’t understand the importance or if they don’t see any signs, they may be left behind” (medical director, age 43), and “since there are no physical symptoms, they see if they live far from the hospital, they decide not to spend the transport fee and conclude that they are fine” (CCS service provider, age 45).

Another interviewee explained that, when asking patients why they delayed the follow-up screening, “they used to mention that they were fine; they observed no symptoms or they forgot their appointment” (CCS focal person, age 34). This statement also emphasized the absence of symptoms as a predicament and additionally introduced forgetting the follow-up appointment as a common explanation for missing or delaying the follow-up screening. A perception that was shared by another interviewee who said, when asking his clients “why they didn’t show up for their follow-up appointment on the scheduled date, the majority of them said they had forgotten” (CCS service provider, age 28).

Finally, a fear of pain during examination or treatment emerged as a recurring motive. One NCD team leader claimed: “The major barrier [is] the fear the clients have towards the metal speculum. Some of them even after taking the screening for the first time won’t come again for follow-up since they remember the procedure with the metal speculum” (age 30). Another participant shared: “When we conduct cryotherapy, there might be some kind of pain or bleeding in some cases, so due to the fear of such kinds of symptoms, they didn’t want to receive the treatment” (NCD team leader, age 29). In 1 specific case, when an NCD team leader asked a client for her reasons not to come back for the follow-up screening, “the answer was fear of the pain of the [cryotherapy] process” (age 29).

### Health facility-related barriers

The second group of barriers discussed during the IDIs was factors related to the structure and equipment of the health facilities, and on a grander scale, the healthcare system. One central topic was poor counseling by healthcare providers. Interview partners expressed the belief that clients are willing to follow the advice given by healthcare professionals but often do not receive adequate counseling. For example, 1 NCD team leader stated: “From my experience, our community believes and accepts advice from health professionals if well informed. But if in-depth counseling is not given, and if it is given in a rush, it could be one reason” (age 52). Some of the health professionals argued that poor counseling was related to an insufficient number of healthcare providers. A medical director said: “[Patients do not] get good counseling due to the lack of health care providers in cervical cancer screening units. […] There are only two healthcare providers there. Even there was one previously. There is a lack of time, so I don’t think they give attention to counseling” (age 36).

According to most of the interviewed partners, the shortage of trained staff did not only affect the quality of the counseling but also the provision of the follow-up screening itself. As pointed out in the following remark, most health centers employed only 1 person providing CCS, without a backup: “In most health facilities, in health centers that have a shortage of personnel, there is only one trained health care provider. Most of the time, they are nurses or midwives. When the nurse or midwife placed there has night duty and becomes the day off, the clients lose the service due to this” (CCS service provider, age 35). Furthermore, the participants explained that the health professionals providing CCS were often required to assist in other parts of the health facility and were therefore unable to provide the service. Examples included: “Due to different campaign programs like vaccination programs which include polio and COVID-19 vaccination[…]the precancerous cervical cancer screening program may halt the service for the duration of the campaign work” (CCS service provider, age 28), and “during these times [when I am working on another hospital task], this room is closed; mothers won’t be able to find me, and as a result, they leave without being rescreened” (CCS service provider, age 45). Especially those clients who came to the health center for screening but did not encounter anyone, there were often lost to follow-up.

Another frequently mentioned issue was the insufficient reminder system. While many health professionals described solely relying on appointment cards, only a minority said they called patients regularly. The reasons for not being able to call clients touched on different aspects. Lacking the necessary telephone was pointed out by 1 NCD team leader: “The first thing that I want to mention in this area is that health professionals in the department didn’t make phone calls as reminders for follow-up examinations. Maybe they have different reasons for that including a lack of office telephone access and a mobile card” (age 29). One CCS provider even described using her private phone to make the phone calls: “In my previous experience, I called clients as a reminder for their appointment date, and they came for their follow-up. But later, they saved my phone number and called me outside of working hours, including midnight, just to discuss other medical conditions” (age 32). Some participants also related the deficient reminder system with the shortage of staff: “Since only one health care provider is working here, there is a problem with tracking and calling appointed women. […] If there are two healthcare providers, it may be easier to select post-treatment follow-up mothers or untreated mothers and make calls to remind them” (CCS service provider, age 29)

A third aspect mentioned regularly in relation to the reminder system was the quality of the documentation. Difficulties regarding the logbook documentation were described by this CCS service provider: “I occasionally come across documentation issues such as illegible handwriting, unregistered phone numbers, incorrectly addressed names, and missing appointment dates” (age 30). Additionally, frequent changes of the logbook format were mentioned: “Sometimes, it [the logbook] is changed after we use only the first page. It is extra work to find the old one. […] If it is changed within months or weeks, we lose mothers in between because we don’t focus on the old logbooks” (CCS service provider, age 29).

### Actions to be taken by responsible bodies of the healthcare system

**Figure 2.**
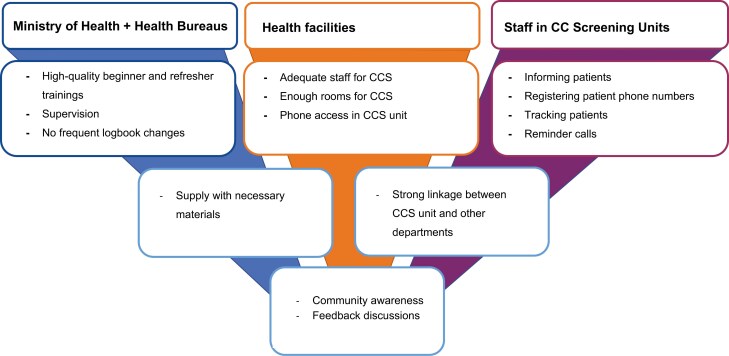
Recommended actions by responsible bodies in the healthcare system to improve cervical cancer screening follow-up adherence mentioned by the health professionals.

The IDI participants suggested different strategies (Figure 2) for all involved stakeholders to address the given barriers across all levels of the healthcare system to subsequently improve the adherence to follow-up. The stakeholders include the MoH, health bureaus, health facilities, and health professionals who work in CCS departments. The MoH is responsible for providing high-quality beginner and refresher trainings for CCS and cryotherapy; in those trainings, the importance of follow-up and in-depth counseling should be stressed, and guideline adherence promoted. Subsequently, the MoH might offer a system of supervision and allow feedback discussions with health professionals and health facilities. Together with the health facilities, the MoH should ensure that the materials necessary for CCS and treatment are available. Besides those materials, health facilities should provide rooms that are easy to locate and allow appropriate privacy while conducting CCS as well as phone access for reminder calls in all CCS units. What is more, some of the interviewed staff shared their disappointment about missing linkage with other departments within the same health facility, such as gynaecology, out patient department (OPD), and HIV departments. The health professionals providing CCS should uphold a high standard of care, provide good information and counseling, track women in need of follow-up, and make reminder calls. Finally, all stakeholders were encouraged to work together to raise community awareness.

## Discussion

In this study, the most common reasons given by women who did not attend rescreening 1 year after treatment for precancerous lesions were not being aware of the follow-up, forgetfulness, perceiving follow-up as not needed, feeling healthy, moving to another place, being temporarily away, and lack of time. The findings from the IDIs conducted with health professionals aligned around barriers to posttreatment follow-up, including lack awareness, forgetfulness, poor health-seeking behavior, residency-related barriers, and lack of time due to household responsibilities. Moreover, the health professionals reported health-facility-related barriers such as a shortage of trained healthcare providers, a poor counseling service, and the lack of a reminder system.

The telephone participants mentioned not being aware of the follow-up as a common reason for not adhering to follow-up recommendations. A cohort study conducted in Cameroon also reported the lack of information as a main barrier to rescreening.^24^ Most of our IDI participants also agreed that the lack of awareness was one of the main barriers to women’s adherence to their posttreatment follow-up. Poor health-seeking behavior was another barrier discussed. Health professionals stressed that women might think that there is no need to go to the health facility if they do not feel sick, which can be explained by a lack of awareness and poor understanding of the importance of posttreatment follow-up. Studies conducted in southwest Nigeria,^25^ the United States,^26^ and a multicountry research project in Bolivia, Peru, Kenya, South Africa, and Mexico^20^ also support this finding. This issue can probably be minimized by effective counseling.

Conversely, poor counseling services were cited as a barrier. This is supported by a qualitative study in Cameroon that found inadequate counseling as a barrier to follow-up of precancerous lesion treatment.^21^ A study in Jamaica showed that women who were given advice on follow-up timing were 6 times more likely to seek appropriate follow-up.^27^ Other studies also showed that adherence to follow-up was associated with effective communication, being informed of their screening results, and having correct awareness of the outcome.^28^ Hence, counseling can serve as a tool for educating patients on the necessity of follow-up procedures, addressing fear, providing emotional support, and overcoming barriers. Adequate and culturally adaptive counseling should be given to patients to improve adherence to follow-up.

Forgetting the appointment was mentioned by both the patients and health professionals as one of the main reasons for the loss of follow-up. This might be due to patients’ lack of attention due to other responsibilities or the lack of a reminder. These findings align with a study based on a review of several studies showing “forgetting appointments” as one of the most common barriers to follow-up.^28^ Apart from good counseling, reminder systems could be one effective way of diminishing this barrier; the Ethiopian cervical cancer prevention and control guideline recommends “telephone women at home or at work” and “health extension workers and case managers to contact women directly at home.”^5^ A study in Honduras found that reminder phone calls were highly successful at recalling women for HPV retesting.^29^ Another study conducted in Kenya revealed that sending SMS reminders for revisits resulted in a 5-fold rise in the number of patients who received clinically appropriate care following a positive screening result.^30^ The Tanzanian study also found that the majority of HPV-positive women attended their follow-up appointment after receiving a text message.^31^ However, despite the guideline suggesting to telephone women who do not return for follow-up, in the IDIs, it became clear that these reminder systems are not yet well implemented due to various reasons, including missing telecommunication equipment, difficulties in tracking eligible patients due to frequent changes of screening logbooks, and a shortage of staff.

Staff shortage was also discussed as a key problem in providing the required services for follow-up; in particular, the absence of healthcare providers conducting the screening during operation hours, insufficient personnel to ensure adequate documentation, and a lack of time to provide adequate counseling posed as major challenges. Problems with trained staff not working at respective sites and additional responsibilities of service providers other than CCS and treatment were already identified in the health facility assessment of the Addis tesfa project in Ethiopia.^14^ A study that summarizes the experiences of research projects in different countries came to similar conclusions regarding staff shortages and their effect on follow-up.^20^

Another identified barrier was the fear of pain during an examination or therapy. This finding is consistent with a review of several studies that identified “fear of diagnosis and treatment” as one of the obstacles to follow-up.^28^ Some people may skip rescreenings out of fear of experiencing pain. Severe pain during these procedures is not generally anticipated. Women who experience severe pain on their first visit should receive extra attention and counseling for the following visits, and it is critical for patients to express their fears and discomfort in order to receive appropriate support and treatment.

During the IDIs, geographical distance from the health facility, transport costs, and changing the place of residence were frequently discussed as patient-related barriers to adhering to a follow-up screening. A study in Honduras also mentioned moving away from the clinic area as a barrier to follow-up adherence.^29^

Another barrier in this study was the husband’s influence on the health-seeking behavior of the clients, in line with studies in Cameroon^21^ and Mexico^20^ that showed male partners’ influence and lack of support to attend follow-up visits as barriers to follow-up. In another study conducted in rural Lilongwe, Malawi, male companions were mentioned as both barriers and valuable sources of support including encouragement, emotional support, and assistance in overcoming transportation obstacles.^32^ The clients’ responsibilities such as childcare were also discussed in our study. Research to identify hurdles to follow-up in Latina women with abnormal Pap smears who were referred for colposcopy also identified childcare responsibility as a barrier.^26^

### Strengths and limitations

To our knowledge, this mixed-methods study was the first of its kind to give insight into barriers to adherence to follow-up recommendations in Addis Ababa and the Oromia region. The findings from both methods complement each other, including perspectives from different health professionals that allow for a broad picture of the health facility-related barriers. However, IDIs were conducted only with healthcare providers since we were unable to interview patients in person. We tried to tackle this issue by addressing reasons for not attending follow-up during the quantitative telephone interviews.

## Conclusion

This mixed-methods study assessed various barriers to follow-up after treatment for cervical precancerous lesions in Ethiopia. We identified that the lack of a patient tracking system and a lack of reminders contributed to the low uptake of rescreening. Forgetfulness, fear, lack of awareness, poor health-seeking behavior, residency-related barriers like living in a remote area or changing living area, and socioeconomic barriers such as lack of time due to household responsibilities, the husband’s influence, and a lack of money to travel were identified as patient-related barriers, while a shortage of trained healthcare providers, poor counseling, and an insufficient reminder system were the health facility-related barriers to follow-up adherence.

Effective interventions, such as creating community awareness, improving patient counseling, improving the system of tracing patients in need of follow-up, and making reminder calls should be targeted by different stakeholders to tackle these barriers. In line with efforts to up-scale the digitalization of health systems, patients should also directly benefit. Health workers could directly inform patients 1 year after the treatment of suspicious cervical findings, for instance, through SMS reminders. Given the massive governmental efforts in Ethiopia to offer primary screening, rescreening compliance in high-risk patients should be a priority to ensure the success of the whole program.

## Supplementary material

Supplementary material is available at *The Oncologist* online.

oyae305_suppl_Supplementary_Material_1

oyae305_suppl_Supplementary_Material_2

## Data Availability

Due to confidentiality, the data are not available publicly but can be made available upon reasonable request to the first author.

## References

[1] World Health Organization. Cervical cancer [Internet]. Accessed November 9, 2021. https://www.who.int/westernpacific/health-topics/cervical-cancer

[2] Rerucha CM , Caro RJ, Wheeler VL. Cervical cancer screening. Am Fam Physician. 2018;97:441-448. 29671553

[3] Perkins RB , Guido RS, Castle PE, Chelmow D, Einstein MH, Garcia F, et al 2019 ASCCP Risk-Based Management Consensus Guidelines for abnormal cervical cancer screening tests and cancer precursors. J Low Genit Tract Dis. 2020;24:102-131. https://doi.org/10.1097/LGT.0000000000000525 32243307 PMC7147428

[4] Bruni L , Albero G, Serrano B, et al ICO/IARC Information Centre on HPV and Cancer (HPV Information Centre). Human Papillomavirus and Related Diseases in Ethiopia. Summary Report October 22, 2021.

[5] Federal Democratic Republic of Ethiopia Ministry of Health. Guideline for cervical cancer prevention and control in Ethiopia, April 2021.

[6] Kocken M , Helmerhorst TJM, Berkhof J, et al Risk of recurrent high-grade cervical intraepithelial neoplasia after successful treatment: a long-term multi-cohort study. Lancet Oncol. 2011;12:441-450. https://doi.org/10.1016/S1470-2045(11)70078-X 21530398

[7] Adam Y , Gelderen CJ van, Bruyn G de, et al Predictors of persistent cytologic abnormalities after treatment of cervical intraepithelial neoplasia in Soweto, South Africa: a cohort study in a HIV high prevalence population. BMC Cancer. 2008;8:211. 18657270 10.1186/1471-2407-8-211 PMC2515323

[8] Zeier MD , Nachega JB, Van Der Merwe FH, et al Impact of timing of antiretroviral therapy initiation on survival of cervical squamous intraepithelial lesions: a cohort analysis from South Africa. Int J STD AIDS. 2012;23:890-896. https://doi.org/10.1258/ijsa.2012.012040 23258831

[9] Oga EA , Brown JP, Brown C, et al Recurrence of cervical intraepithelial lesions after thermo-coagulation in HIV-positive and HIV-negative Nigerian women. BMC Womens Health. 2016;16:25. https://doi.org/10.1186/s12905-016-0304-8 27169666 PMC4864941

[10] Bogale AL , Teklehaymanot T, Ali JH, Kassie GM, Medhin G. The recurrence of cervical precancerous lesion among HIV positive and negative Ethiopian women after cryotherapy: a retrospective cohort study. Cancer Control J Moffitt Cancer Cent [Internet]. 2022;29:10732748221129708. [cited February 29, 2024]. https://doi.org/10.1177/10732748221129708 PMC9513568 36151596

[11] Phongsavan K , Phengsavanh A, Wahlström R, Marions L. Safety, feasibility, and acceptability of visual inspection with acetic acid and immediate treatment with cryotherapy in rural Laos. Int J Gynaecol Obstetrics. 2011;114:268-272. https://doi.org/10.1016/j.ijgo.2011.03.009 21752376

[12] Barchi F , Winter SC, Ketshogile FM, Ramogola-Masire D. Adherence to screening appointments in a cervical cancer clinic serving HIV-positive women in Botswana. BMC Public Health. 2019;19:318. https://doi.org/10.1186/s12889-019-6638-z 30885175 PMC6423763

[13] Vet JNI , Kooijman JL, Henderson FC, et al Single-visit approach of cervical cancer screening: see and treat in Indonesia. Br J Cancer. 2012;107:772-777. https://doi.org/10.1038/bjc.2012.334 22850550 PMC3425980

[14] Shiferaw N , Salvador-Davila G, Kassahun K, et al The single-visit approach as a cervical cancer prevention strategy among women with HIV in Ethiopia: successes and lessons learned. Glob Health Sci Pract. 2016;4:87-98. https://doi.org/10.9745/GHSP-D-15-00325. 27016546 PMC4807751

[15] World Health Organization, International Agency for Research on Cancer, African Population and Health Research Center. Prevention of cervical cancer through screening using visual inspection with acetic acid (VIA) and treatment with cryotherapy. A demonstration project in six African countries: Malawi, Madagascar, Nigeria, Uganda, the United Republic of Tanzania, and Zambia [Internet]. World Health Organization; 2012. iv, 33 p. Accessed December 7, 2021. https://apps.who.int/iris/handle/10665/75250

[16] Stroetmann CY , Gizaw M, Alemayehu R, et al Adherence to treatment and follow-up of precancerous cervical lesions in Ethiopia. Oncologist. 2024;29:e655-e664. https://doi.org/10.1093/oncolo/oyae027 38394385 PMC11067800

[17] Peterson NB , Han J, Freund KM. Inadequate follow-up for abnormal Pap smears in an urban population. J Natl Med Assoc. 2003;95:825-832. 14527050 PMC2594474

[18] Sharp L , Cotton S, Thornton A, et al Who defaults from colposcopy? A multi-centre, population-based, prospective cohort study of predictors of non-attendance for follow-up among women with low-grade abnormal cervical cytology. Eur J Obstet Gynecol Reprod Biol. 2012;165:318-325. https://doi.org/10.1016/j.ejogrb.2012.08.001 22921577

[19] Kiptoo S , Otieno G, Tonui P, et al Loss to follow-up in a cervical cancer screening and treatment program in Western Kenya. J Global Oncol. 2018;4:97s-97s. https://doi.org/10.1200/jgo.18.41300

[20] Bingham A , Bishop A, Coffey P, et al Factors affecting utilization of cervical cancer prevention services in low-resource settings. Salud Pública de México. 2003;45:408-416. https://doi.org/10.1590/s0036-36342003000900015 14746034

[21] Manga S , Kiyang E, DeMarco RF. Barriers and facilitators of follow-up among women with precancerous lesions of the cervix in Cameroon: a qualitative pilot study. Int J Womens Health. 2019;11:229-239. https://doi.org/10.2147/IJWH.S196112 31015770 PMC6448541

[22] Qcamap.org [Internet]. Accessed April 24, 2021. https://www.qcamap.org/ui/en/home

[23] Mayring P. Qualitative Content Analysis : *Theoretical Foundation, Basic Procedures and Software Solution*. 2014:143.

[24] Evina Bolo S , Kenfack B, Wisniak A, et al Factors influencing cervical cancer re-screening in a semi-rural health district of Cameroon: a cohort study. BMC Womens Health. 2024;24:76. https://doi.org/10.1186/s12905-024-02917-3 38281960 PMC10822157

[25] Ezechi OC , Petterson KO, Gabajabiamila TA, et al Predictors of default from follow-up care in a cervical cancer screening program using direct visual inspection in south-western Nigeria. BMC Health Serv Res. 2014;14:143. 24678898 10.1186/1472-6963-14-143 PMC3986612

[26] Percac-Lima S , Aldrich LS, Gamba GB, Bearse AM, Atlas SJ. Barriers to follow-up of an abnormal Pap smear in Latina women referred for colposcopy. J Gen Intern Med. 2010;25:1198-1204. https://doi.org/10.1007/s11606-010-1450-6 20652647 PMC2947627

[27] Jeong SJ , Saroha E, Knight J, Roofe M, Jolly PE. Determinants of adequate follow-up of an abnormal Papanicolaou result among Jamaican women in Portland, Jamaica. Cancer Epidemiol. 2011;35:211-216. https://doi.org/10.1016/j.canep.2010.07.004 20688592 PMC3062074

[28] Khanna N , Phillips MD. Adherence to care plan in women with abnormal Papanicolaou smears: a review of barriers and interventions. J Am Board Fam Pract. 2001;14:123-130. 11314919

[29] Thomson KA , Sandoval M, Bain C, et al Recall efforts successfully increase follow-up for cervical cancer screening among women with Human Papillomavirus in Honduras. Glob Health Sci Pract. 2020;8:290-299. https://doi.org/10.9745/GHSP-D-19-00404 32606095 PMC7326516

[30] Mabachi NM , Wexler C, Acharya H, et al Piloting a systems level intervention to improve cervical cancer screening, treatment and follow up in Kenya. Front Med. 2022;9:930462. https://doi.org/10.3389/fmed.2022.930462 PMC9520305 36186820

[31] Mremi A , Linde DS, Mchome B, et al Acceptability and feasibility of self-sampling and follow-up attendance after text message delivery of human papillomavirus results: a cross-sectional study nested in a cohort in rural Tanzania. Acta Obstet Gynecol Scand. 2021;100:802-810. https://doi.org/10.1111/aogs.14117 33555038

[32] Chapola J , Lee F, Bula A, et al Barriers to follow-up after an abnormal cervical cancer screening result and the role of male partners: a qualitative study. BMJ Open. 2021;11:e049901. https://doi.org/10.1136/bmjopen-2021-049901 PMC8442050 34521669

