# Supplementary material for: Barriers to adherence of posttreatment follow-up after positive primary cervical cancer screening in Ethiopia: a mixed-methods study
Source: Oncologist. 2024 Nov 18;30(7):oyae305. doi: 10.1093/oncolo/oyae305 (PMC12311284; doi:10.1093/oncolo/oyae305)
Supplement: oyae305_suppl_Supplementary_Material_1 [file oyae305_suppl_supplementary_material_1.docx]

# INTERVIEW GUIDE (ENGLISH VERSION)

Date of In-depth interview: ____/______/________________

Name of Note Taker: ___________________________

Start time:

End time:

INFORMATION ON THE INTERVIEWEE

Age in years: ______

Sex: ○ MALE ○ FEMALE

Responsibility/position in the clinic: _____________________________

Work experience on the position: _____________________________

Have you ever received training for cervical cancer screening?
 ○ YES ○ NO

Have you ever received training for cryotherapy/?
 ○ YES ○ NO

IN-DEPTH INTERVIEW

1. Please, tell me about your experiences giving service for women who have suspicious cervical lesions in VIA screening.
2. How do you see the skills of the health professionals working in the health facility? Are they able to screen-and-treat?

**Probe:** Training and supervision programs?

1. How do you describe the level of adherence to follow-up among patients, one year after treatment for precancerous cervical lesions?

Probe: Please estimate, how many of patients come one year after treatment for precancerous cervical lesions.

What do you think are possible reasons for loss of follow-up?

1. What actions are you taking after treatment with cryotherapy?

**Probe:** How much emphasis do you give for follow-up recommendation?

Follow-up reminders?

Link with health extension workers?

1. What are barriers to precancerous cervical lesions post-treatment follow-up adherence do you see in general?

**Probe:** Barriers to precancerous cervical lesions post-treatment follow-up adherence in your facility?

1. Please tell me about the documentation in the cervical cancer screening logbook in your health facility?

**Probe:** Do you report adherence to follow-up?

New policies /guidelines? Training?

1. What do you recommend for improvement of post-treatment follow-up adherence?
