# Supplementary material for: Barriers to adherence of posttreatment follow-up after positive primary cervical cancer screening in Ethiopia: a mixed-methods study
Source: Oncologist. 2024 Nov 18;30(7):oyae305. doi: 10.1093/oncolo/oyae305 (PMC12311284; doi:10.1093/oncolo/oyae305)
Supplement: oyae305_suppl_Supplementary_Material_2 [file oyae305_suppl_supplementary_material_2.docx]

### Supplement 2: Themes, subthemes and categories of barriers to follow-up

| **Themes** | **Subthemes** | **Categories** |
| --- | --- | --- |
| Patient-related barriers | Socio-economic barriers | - Household responsibilities/lack of time - Not getting husbands permission - Lack of travel cost |
|  | Lack of awareness/knowledge | - Lack of awareness - Not understanding the importance |
|  | Poor health seeking behavior | - Denial - Feeling healthy - Not giving attention for prevention |
|  | Residency related barriers | - Living in distant/remote areas - Changing living area - Being non-permanent resident |
|  | Forgetfulness | - Forgetting |
|  | Fear | - Fear of screening procedure - Fear of speculum - Fear of metal speculum - Fear of pain - Fear of treatment pain |
| Health facility related barriers | Shortage of trained health care provider | - Lack of health care provider - Additional tasks on the health care provider - Unavailability of the health care provider during their visit |
|  | Poor counseling and approach during the first visit | - Poor counseling - Bad reception at the former visit |
|  | Reminder related barriers | - Lack of staff to facilitate calling to remind - Not calling to remind - Lack of telephone in the unit - Frequent change of logbook - Improper documentation of patient data - Non-functional phone numbers |
